# Interleukin-23 Restrains Regulatory T Cell Activity to Drive T Cell-Dependent Colitis

**DOI:** 10.1016/j.immuni.2008.02.019

**Published:** 2008-04-11

**Authors:** Ana Izcue, Sophie Hue, Sofia Buonocore, Carolina V. Arancibia-Cárcamo, Philip P. Ahern, Yoichiro Iwakura, Kevin J. Maloy, Fiona Powrie

**Affiliations:** 1 Sir William Dunn School of Pathology, University of Oxford, South Parks Road, Oxford OX1 3RE, United Kingdom; 2 Center for Experimental Medicine, Institute of Medical Science, University of Tokyo, Tokyo 108-8639, Japan

**Keywords:** CELLIMMUNO

## Abstract

Interleukin-23 (IL-23) is an inflammatory cytokine that plays a key role in the pathogenesis of several autoimmune and inflammatory diseases. It orchestrates innate and T cell-mediated inflammatory pathways and can promote T helper 17 (Th17) cell responses. Utilizing a T cell transfer model, we showed that IL-23-dependent colitis did not require IL-17 secretion by T cells. Furthermore, IL-23-independent intestinal inflammation could develop if immunosuppressive pathways were reduced. The frequency of naive T cell-derived Foxp3^+^ cells in the colon increased in the absence of IL-23, indicating a role for IL-23 in controlling regulatory T cell induction. Foxp3-deficient T cells induced colitis when transferred into recipients lacking IL-23p19, showing that IL-23 was not essential for intestinal inflammation in the absence of Foxp3. Taken together, our data indicate that overriding immunosuppressive pathways is an important function of IL-23 in the intestine and could influence not only Th17 cell activity but also other types of immune responses.

## Introduction

Identifying tissue-specific factors that control the immune response is important for designing targeted therapies. Indeed, there is increasing recognition that regional immune responses can involve distinct effector pathways and mechanisms of control that may be different from the systemic immune response. Recent studies have highlighted the role of IL-23 as an important mediator of tissue inflammatory responses.

Interleukin-23 (IL-23) is a member of the IL-12 family of heterodimeric cytokines. It is composed of IL-12p40, which is common to IL-12, and the IL-23-specific p19 subunit (Kastelein et al., 2007; Oppmann et al., 2000). IL-23 has been shown to be important in a number of inflammatory diseases including experimental autoimmune encephalitis (EAE), collagen-induced arthritis (CIA), colitis, and dermal inflammation (Cua et al., 2003; Murphy et al., 2003; Uhlig et al., 2006b; Zheng et al., 2007). In various models of intestinal inflammation, IL-23 has been shown to be preferentially expressed in the intestine rather than in the spleen, suggesting a tissue-specific function (Hue et al., 2006; Uhlig et al., 2006b).

In humans, increased amounts of IL-23 have been associated with rheumatoid arthritis, multiple sclerosis, and psoriasis (Kim et al., 2007; Lee et al., 2004; Vaknin-Dembinsky et al., 2006). However, the relevance of IL-23 in human disease is highlighted by the finding that polymorphisms within the *IL23R* gene locus are linked to susceptibility to the two forms of inflammatory bowel disease (IBD), Crohn's disease (CD), and ulcerative colitis (UC) (Duerr et al., 2006). Interestingly, that study also identified an uncommon allele of the *IL23R* that confers protection against CD. This large-scale study was further confirmed by an independent genome-wide analysis (Wellcome Trust Case Control Consortium, 2007). In addition to *IL23R*, this latter study also identified a linkage of CD to a region containing *STAT3*, a mediator of IL-23 signaling. Together, these data strongly suggest a role for IL-23 in human IBD. This finding correlates with results in mouse models showing that IL-23 plays a key role in driving intestinal inflammation mediated by innate immune cells and T cells (Elson et al., 2007; Hue et al., 2006; Kullberg et al., 2006; Uhlig et al., 2006b; Yen et al., 2006).

The effects of IL-23 on CD4^+^ T cells have mainly been linked to the T helper 17 (Th17) cell response. Th17 cells are a recently described T cell subset driven by the transcription factor RORγt (Ivanov et al., 2006). They appear to mediate host protective immunity to some extracellular bacteria and fungi and can mount potent inflammatory responses in several mouse models of autoimmunity, such as EAE and CIA (Weaver et al., 2007). Because IL-23-deficient mice are resistant to both diseases, it was initially thought that IL-23 was necessary for Th17 differentiation (Harrington et al., 2005; Langrish et al., 2005; Park et al., 2005). However, subsequent studies indicated that Th17 differentiation was dependent on TGF-β and IL-6 or IL-21, whereas IL-23 could act by reinforcing the Th17 response (Bettelli et al., 2006; Korn et al., 2007; Mangan et al., 2006; Nurieva et al., 2007; Veldhoen et al., 2006; Zhou et al., 2007). Thus, although IL-23 appears to sustain Th17 responses in vivo, it is not required for Th17 polarization in vitro. Elucidation of the role of IL-23 in the generation and/or expansion of Th17 has further been hampered by the fact that naive T cells from spleen and blood do not seem to express the IL-23R, which appears to be induced after T cell activation in the presence of IL-6 or IL-21 (Ivanov et al., 2006; Zhou et al., 2007).

Although the focus of many studies has been on the IL-23-Th17 axis, there is evidence pointing to a role for IL-23 on T cells that is independent of IL-17 production. Thus IL-23p19-deficient mice are resistant to EAE, whereas IL-17-deficient mice are susceptible to the disease, albeit with a delayed onset and reduced severity (Cua et al., 2003; Komiyama et al., 2006). Furthermore, deficiencies in IL-23 lead to susceptibility to infection with the intestinal pathogen *C. rodentium* despite unimpaired induction of a Th17 response (Mangan et al., 2006). Similarly, anti-IL-17 treatment had little impact on the T cell-mediated colitis that develops in IL-10-deficient mice or in RAG-deficient recipients of IL-10-deficient CD4^+^ T cells, although the colitis was dependant on IL-23 (Yen et al., 2006).

Despite the importance of IL-23 in IBD, there remains a lack of conclusive data on how it functions to promote T cell-dependent colitis. Here, we have assessed T cell-mediated inflammation in a mouse model of colitis in the presence or absence of IL-23. Unexpectedly, our results demonstrate that IL-23 reduces the frequency of Foxp3^+^ cells in the intestine and that in the absence of regulatory T (Treg) cells, IL-23 is dispensable for intestinal inflammation.

## Results

### T Cell-Derived IL-17 Is Not Required for Intestinal Inflammation

**Figure 1:**
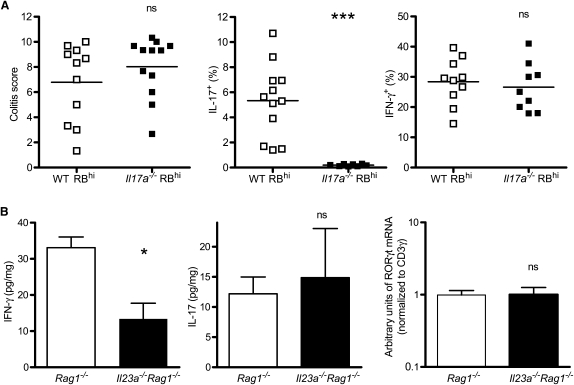
T Cell-Derived IL-17 Is Not Essential for Colitis (A) Transfer of *Il17a^−/−^* CD4^+^CD45RB^hi^ T cells into *Rag1^−/−^* mice. Left: colitis scores for recipients transferred with wild-type or IL-17-deficient CD4^+^CD45RB^hi^ T cells. Each point represents an individual mouse. Data are representative of four independent experiments; graph shows pooled data from two independent experiments. Center and right: Percentage of IL-17^+^ (center) or IFNγ^+^ (right) cells among CD4^+^ cells isolated from the colonic lamina propria from the mice analyzed left. (B) Characterization of Th17 and Th1 cell responses in the absence of IL-23. Amounts of IFN-γ (left) and IL-17 (center) in colon homogenates of *Rag1^−/−^* or *Il23a^−/−^Rag1^−/−^* mice transferred with wild-type naive T cells. Right: Amounts of RORγt mRNA in colon homogenate. Values are normalized to CD3γ expression. Data show mean + SEM of between five and ten mice from two independent experiments. ^∗^, p < 0.05; ^∗∗∗^, p < 0.001.

To dissect the role of IL-23 in colonic inflammation, we used a well-characterized mouse model of colitis. In this model, naive CD4^+^ CD45RB^hi^ T cells transferred into immunodeficient hosts react to the intestinal flora to induce IL-23-dependent colonic inflammation (Hue et al., 2006; Powrie et al., 1993). Because IL-23 promotes IL-17 production by CD4^+^ T cells, we reasoned that colitis might be dependent on T cell-derived IL-17. However, IL-17-deficient T cells are not impaired in their ability to induce colitis (Noguchi et al., 2007) (Figure 1A). The percentage of IFN-γ-producing T cells in the intestine remains unaffected (Figure 1A), indicating that the inflammation induced by *Il17a^−/−^* T cells is not due to a compensatory increase in Th1 cells.

We next assessed the effect of IL-23 on intestinal IL-17 upon T cell transfer. Unlike IFN-γ, which was decreased in the colons of IL-23-deficient recipients, the amount of IL-17 was unaffected by the absence of IL-23 (Figure 1B), despite the fact that *Il23a^−/−^Rag1^−/−^* recipients did not develop intestinal inflammation (data not shown). Likewise, lack of IL-23 did not significantly affect the relative amounts of the Th17-specific factor RORγt in the colon (Figure 1B). Together, these data suggest that Th17 cell responses are not specifically impaired in the intestine of IL-23-deficient mice and point to effects of IL-23 beyond Th17 promotion.

### IL-23-Independent Intestinal Inflammation in the Absence of IL-10 or TGF-β

**Figure 2:**
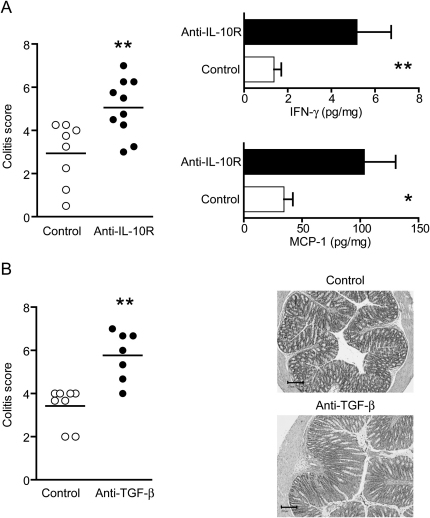
Reduction of Regulatory Pathways Increases Colitis in *Il23a^−/−^Rag1^−/−^* Mice Transferred with Naive T Cells (A) Blockade of the IL-10 pathway after transfer of CD4^+^CD45RB^hi^ T cells into *Il23a^−/−^Rag1^−/−^* mice. Left: Colitis scores for control untreated and anti-IL-10R-treated recipients. Each point represents an individual mouse. Right: Concentration of proinflammatory cytokines (mean + SEM) in colon homogenates from these mice. (B) Blockade of TGF-β after transfer of CD4^+^CD45RB^hi^ T cells into *Il23a^−/−^Rag1^−/−^* mice. Left: Colitis scores for control untreated and anti-TGF-β treated recipients. Each point represents an individual mouse. Right: Representative microphotographs of colonic sections from either control untreated recipients (score 3) or recipients treated with blocking TGF-β antibody (score 6). The scale bars represent 200 μm. Data are pooled from two independent experiments. ^∗^, p < 0.05; ^∗∗^, p < 0.01.

Inflammation is the outcome of a dynamic equilibrium between activating and inhibitory signals. We reasoned that the ablation of IL-23 may shift the equilibrium toward immune suppression, which could abrogate the existing proinflammatory signals. IL-10 has been shown to play an important role in intestinal homeostasis; therefore, we used a blocking IL-10R monoclonal antibody to reveal the presence of pathogenic pathways in *Il23a^−/−^Rag1^−/−^* mice. Upon naive T cell transfer, anti-IL-10R treatment resulted in significantly increased colonic inflammation compared to untreated controls (Figure 2A). Accordingly, the amounts of the proinflammatory cytokines MCP-1 and IFN-γ were increased in colon homogenates isolated from *Il23a^−/−^Rag1^−/−^* mice that had received anti-IL-10R (Figure 2A). To validate this point, we decided to block another intestinal regulatory pathway, the one mediated by TGF-β. TGF-β can also promote inflammation by inducing Th17 cells. However, mice deficient in TGF-β1 die of inflammatory disease (Shull et al., 1992), showing that its anti-inflammatory role is crucial for immune homeostasis. As before, we transferred naive CD4^+^ cells into *Il23a^−/−^Rag1^−/−^* mice and treated the recipients with a blocking TGF-β antibody. As observed for the IL-10 pathway, blocking the TGF-β pathway resulted in a significant increase in the amounts of intestinal inflammation (Figure 2B). Although neither treatment restored the colitis levels observed in IL-23-sufficient RAG-deficient recipients upon T cell transfer (typical colitis score of 8–9), these results suggest that in the absence of IL-23 there is a shift toward IL-10- and TGF-β-mediated immune suppression that masks potential inflammatory pathways.

### T Cells that Cannot Respond to TGF-β Induce Colitis in the Absence of IL-23

**Figure 3:**
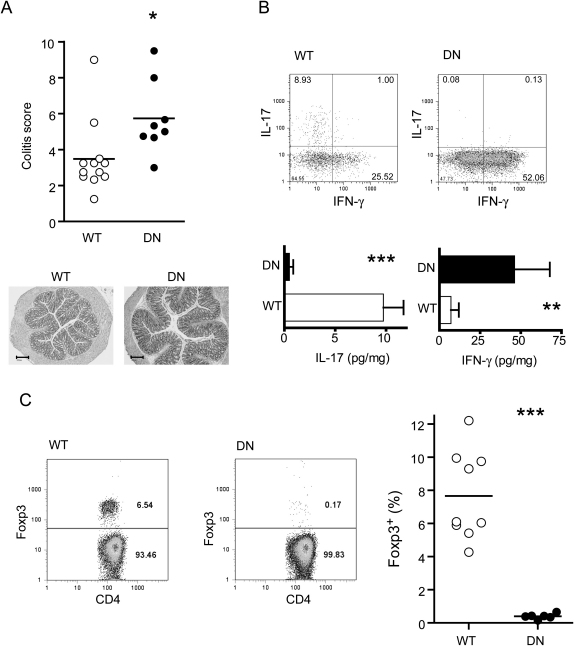
Absence of TGF-β Signaling in T Cells Increases Intestinal Inflammation in *Il23a^−/−^Rag1^−/−^* Mice (A) Colitis score of *Il23a^−/−^Rag1^−/−^* mice transferred with wild-type (WT) or dnTGFβRII (DN) naive T cells. Each point represents an individual mouse. Below: Representative microphotographs of colonic sections from *Il23a^−/−^Rag1^−/−^* mice transferred with WT (score 2) or DN CD4^+^CD45RB^hi^ T cells (score 5). The scale bars represent 200 μm. (B) IL-17 and IFN-γ production in *Il23a^−/−^Rag1^−/−^* mice transferred with wild-type (WT) or dnTGFβRII (DN) naive T cells. The figure shows representative FACS plots of IL-17 and IFN-γ production by MLN lymphocytes gated on CD4^+^ cells and the concentrations of IL-17 and IFN-γ (mean + SEM) in the colon of transferred *Il23a^−/−^Rag1^−/−^* mice. (C) Foxp3 expression in CD4^+^ cells after transfer. Left: Representative FACS plots showing Foxp3 frequency in MLN from transferred *Il23a^−/−^Rag1^−/−^* mice. Plots are gated on CD4^+^ TCRβ^+^ cells. Right: Percentage of Foxp3^+^ among MLN CD4^+^ T cells of transferred *Il23a^−/−^Rag1^−/−^*. Each point represents an individual mouse. Data are pooled from three independent experiments. ^∗^, p < 0.05; ^∗∗^, p < 0.01; ^∗∗∗^, p < 0.001.

We sought to further analyze the regulatory mechanisms that become dominant in the absence of IL-23. TGF-β has been reported to have an immunomodulatory effect on several cell types, including T cells (Li et al., 2006). In order to study the specific effect of TGF-β on T lymphocytes, we used naive T cells isolated from transgenic mice expressing a dominant-negative form of TGF-β receptor II (called dnTGFβRII here) (Gorelik and Flavell, 2000). T cells from these mice have impaired responses to TGF-β signals. In accordance with mAb-blockade of TGF-β, transferred dnTGFβRII T cells induced significant colitis in *Il23a^−/−^Rag1^−/−^* recipients (Figure 3A). This is all the more striking given that dnTGFβRII-naive T cells are less colitogenic than wild-type T cells when transferred into IL-23-sufficient mice (Fahlen et al., 2005) and suggests that TGF-β has predominantly an anti-inflammatory effect on CD4^+^ T cells in the absence of IL-23.

We then ascertained the amounts of IL-17 production by these T cells, a process known to be highly dependent on TGF-β (Bettelli et al., 2006; Mangan et al., 2006; Veldhoen et al., 2006). As expected, we found that it was completely abrogated in cells with impaired responsiveness to TGF-β (Figure 3B). Accordingly, colons from mice transferred with dnTGFβRII T cells showed very low amounts of total IL-17 despite ongoing inflammation, indicating a minor contribution of non-T cell IL-17 in this system. In contrast, the amounts of IFN-γ were significantly increased in the colons of *Il23a^−/−^Rag1^−/−^* mice transferred with dnTGFβRII T cells (Figure 3B). These results suggest that high amounts of IFN-γ might drive the chronic intestinal inflammation in this setting.

Apart from inducing Th17 cells, TGF-β also plays a role in the generation and survival of CD4^+^ Foxp3^+^ regulatory T cells (Chen et al., 2003; Li et al., 2006). When we analyzed the effects of the absence of TGF-β signaling on Foxp3 frequency after naive T cell transfer, we found as expected that this population was almost completely absent among the progeny of dnTGFβRII T cells in the spleen, mesenteric lymph node (MLN), and lamina propria lymphocytes (LPL) (Figure 3C, and data not shown). Interestingly, cells isolated from *Il23a^−/−^Rag1^−/−^* recipients transferred with wild-type naive CD4^+^ T cells contained a sizeable proportion of Foxp3^+^ cells (Figure 3C), considering that the frequency of Foxp3^+^ cells among naive T cells is normally very low. Although the changes in frequency could be due to an increased influx of Foxp3^−^ effector cells in diseased mice, the ratio of regulatory versus pathogenic T cells is key for the inhibition of inflammation. We therefore hypothesized that the FoxP3^+^ population might be related to the resistance to colitis observed in *Il23a^−/−^Rag1^−/−^* mice. We hence assessed whether IL-23 could affect the frequency of Foxp3^+^ cells among the progeny of naive T cells.

### Absence of IL-23 Increases the Frequency of Induced Foxp3^+^CD4^+^T Cells in the Gut

**Figure 4:**
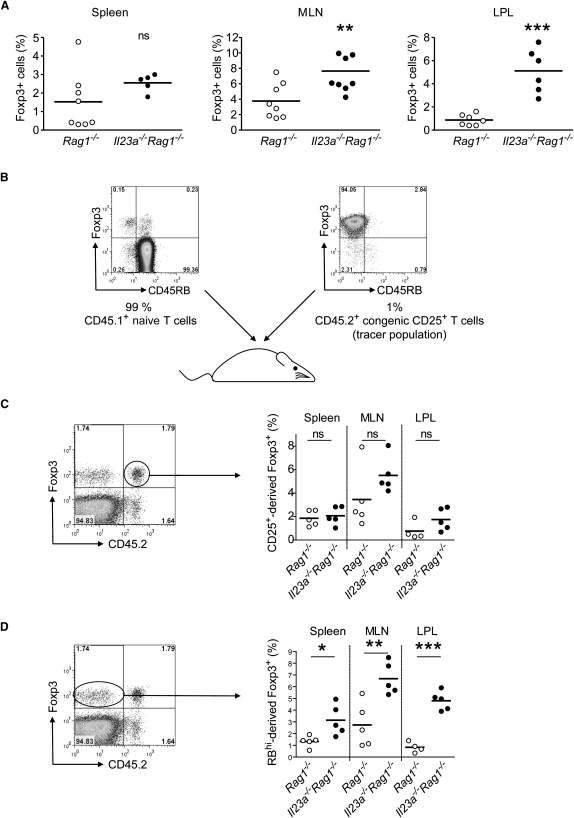
CD45RB^hi^-Derived Foxp3^+^ Cells Are Increased in the Colon after Naive T Cell Transfer into *Il23a^−/−^Rag1^−/−^* Mice (A) Frequency of Foxp3^+^ cells among CD4^+^ T cells from spleen, MLN, and colonic LPL from IL-23-deficient or -sufficient *Rag1^−/−^* recipients transferred with CD4^+^ CD25^−^ CD45RB^hi^ naive T cells. Each point represents an individual mouse; data are pooled from two independent experiments. (B) Design of the congenic-transfer experiment. A mixture of 99% sorted CD45.1^+^ CD4^+^ CD25^−^ CD45RB^hi^ naive T cells and 1% CD45.2^+^ CD4^+^ CD25^+^ CD45RB^low^ regulatory T cells was injected into *Rag1^−/−^* recipients. FACS plots show representative Foxp3 staining of sorted naive (left) and regulatory (right) populations, gated on CD4^+^ cells. (C) Approximately 2 months after transfer, cells from spleen, MLN, and colonic LP were stained for Foxp3 and the congenic marker CD45.2. Left: Representative FACS plot showing Foxp3 versus CD45.2 expression in the spleen of transferred *Il23a^−/−^Rag1^−/−^*. The plot is gated on CD3^+^ CD4^+^ cells. Right: Percentage of CD45.2^+^ Foxp3^+^ cells in the CD3^+^ CD4^+^ population in the spleen, MLN, and LP of transferred IL-23-sufficient or -deficient *Rag1^−/−^* recipients. Each point represents an individual mouse. (D) Percentage of CD45.2^−^ Foxp3^+^ cells in the CD3^+^ CD4^+^ population of the mice analyzed in (C). ^∗^, p < 0.05; ^∗∗^, p < 0.01; ^∗∗∗^, p < 0.001; ns, not significant.

To investigate the effects of IL-23 on the frequency of Foxp3^+^ cells, we compared the percentage of Foxp3^+^ cells after naive T cell transfer into IL-23-sufficient *Rag1^−/−^* or *Il23a^−/−^Rag1^−/−^* recipients. Strikingly, the absence of IL-23 increased the frequency of Foxp3^+^ CD4^+^ T cells in MLN and LPL, whereas no significant increase was observed in the spleen (Figure 4A). Although the frequency of Foxp3^+^ was highest in the MLN from both IL-23-sufficient and -deficient mice, the effect of IL-23 was most pronounced among the lymphocytes isolated from the lamina propria. This correlates with the distribution of IL-23 expression, which is higher in the gut compared to the spleen (Uhlig et al., 2006b).

These data suggest that naive CD4^+^ T cells transferred into an empty host can become Foxp3^+^, but we could not exclude the possibility that a very small number of contaminating Foxp3^+^ T cells proliferate to become a noteworthy population in the absence of IL-23. Although the purity of our naive CD4^+^ CD25^−^ CD45RB^hi^ T cell population was over 99%, technical limitations make it impossible to exclude a contamination of Foxp3^+^ cells of around 0.4%. Moreover, we could also detect a very small population of CD45RB^hi^ Foxp3^+^ cells (Figure 4B), making up around 0.2% of our starting naive population. Given that CD4^+^ Treg cells have been shown to expand when cotransferred with naive T cells into immunodeficient hosts (Izcue et al., 2006), the observed differences could indeed be due to differential accumulation of pre-existing Foxp3^+^ cells in the starting population. To gain a better insight into the origin of the Foxp3^+^ cells that accumulate in the lamina propria of *Il23a^−/−^Rag1^−/−^* recipients, and because we could not eliminate the contaminating Foxp3^+^ population completely, we decided to include a tracer population of preformed Foxp3^+^ cells (Zelenay et al., 2005). To do so, we added 1% of sorted congenic CD45.2^+^ CD4^+^ CD45RB^low^ CD25^+^ cells (Figure 4B) to our CD45.1^+^ naive population. Thus, we had a CD45.2^+^ Foxp3^+^ control population arising from pre-existing CD25^+^ regulatory cells and a CD45.1^+^ Foxp3^+^ population derived from the naive population, which could also include pre-existing Foxp3^+^ cells. When we analyzed the CD25^+^-derived Foxp3^+^ cells in transferred immunodeficient mice, we found no significant differences in the tissue distribution in the presence or absence of IL-23 (Figure 4C). In contrast, Foxp3^+^ cells derived from the naive T cell pool were specifically increased in *Il23a^−/−^Rag1^−/−^* recipients, especially in the colonic lamina propria (Figure 4D). This suggests that contaminating CD4^+^ CD25^+^ Foxp3^+^ do not contribute substantially to the difference in Foxp3^+^ frequencies observed between *Il23a^−/−^Rag1^−/−^* and IL-23-sufficient *Rag1^−/−^* recipients and is consistent with a role for IL-23 in inhibiting the induction of Foxp3 on T cells.

### Foxp3-Deficient Naive T Cells Induce Colitis in the Absence of IL-23

**Figure 5:**
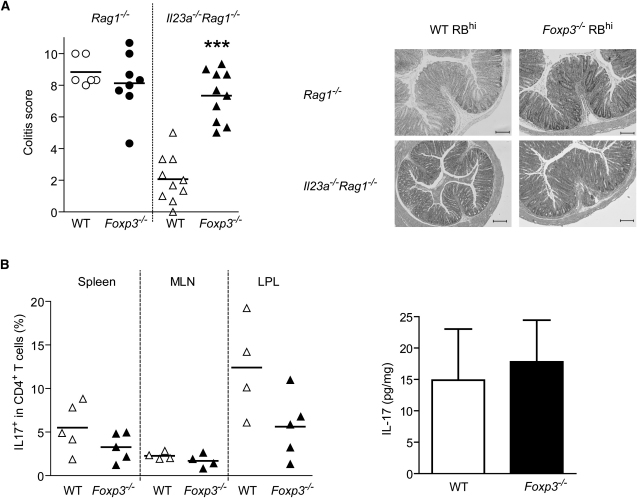
Foxp3-Deficient Naive T Cells Induce Colitis in *Il23a^−/−^Rag1^−/−^* Mice (A) Colitis in mice transferred with Foxp3-deficient naive T cells. Left: Colitis score of *Rag1^−/−^* (circles) or *Il23a^−/−^Rag1^−/−^* (triangles) transferred with wild-type or Foxp3-deficient naive T cells. Each point represents an individual mouse. Right: Representative microphotographs of colonic sections from *Rag1^−/−^* mice transferred with wild-type (score 8) or *Foxp3^−/−^* naive T cells (score 10) or from *Il23a^−/−^Rag1^−/−^* mice transferred with wild-type (score 4) or *Foxp3^−/−^* naive T cells (score 9). The scale bars represent 200 μm. ^∗∗∗^, p < 0.001. (B) Left: Percentage of IL-17-secreting cells in the CD4^+^ population from spleen, MLN, and colon of *Il23a^−/−^Rag1^−/−^* mice transferred with wild-type or *Foxp3^−/−^* naive T cells. Each point represents an individual mouse. Right: Amounts of IL-17 in colon homogenate from *Il23a^−/−^Rag1^−/−^* mice transferred with wild-type or *Foxp3^−/−^* naive T cells. Data show the mean + SEM of nine or ten mice. Differences were not statistically significant. Data are pooled from two independent experiments.

These findings indicated that induction of CD4^+^ Foxp3^+^ T cells may play a role in the control of inflammation in the absence of IL-23. To test this hypothesis, we used T cells isolated from *Foxp3^−/−^* mice (Fontenot et al., 2003). Due to the aberrant T cell activation and early mortality that occur in these mice, it was not possible to use them as a source of naive T cells for transfer experiments. Therefore, mixed bone-marrow chimeras were generated with *Foxp3^−/−^* (CD45.2^+^) and B6.SJL-*Cd45* wild-type congenic CD45.1^+^ donors. Mixed bone-marrow chimeras do not develop the lymphoproliferative pathology that is characteristic of mice deficient in *Foxp3* (Fontenot et al., 2003). Moreover, naive CD4^+^ T cells isolated from these chimeras also contain a small contaminating Foxp3^+^ population, as happens with naive cells isolated from wild-type donors. This provides an additional control for the role of contaminating Foxp3^+^ cells in the starting population. When Foxp3-deficient CD45.2^+^ naive T cells and Foxp3-wild-type CD45.1^+^ naive T cells were isolated and transferred into immunodeficient recipients, Foxp3-deficient cells were able to induce severe colitis in *Il23a^−/−^Rag1^−/−^* recipients (Figure 5A), whereas Foxp3-sufficient cells only induced colitis in IL-23-sufficient *Rag1^−/−^*, but not *Il23a^−/−^Rag1^−/−^*, mice. The level of the inflammation elicited by Foxp3-deficient cells was similar in the presence or absence of IL-23. These results suggest that IL-23 is not essential for T cell-mediated intestinal inflammation, but is key to overriding Treg cell activity.

However, lack of Foxp3 could have an indirect effect on the nature of the immune response. Foxp3^+^ regulatory T cells and Th17 cells appear to be generated through related pathways (Bettelli et al., 2006). Because there is some evidence that Foxp3 can inhibit RORγt-induced IL-17 expression (Ivanov et al., 2006), we wanted to check that the inflammation induced by Foxp3-deficient T cells was not due to a skew toward the Th17 cell phenotype. As shown in Figure 5B, the absence of functional Foxp3 on naive T cells did not lead to an increased frequency of Th17 cells after transfer into *Il23a^−/−^Rag1^−/−^* hosts. Similar frequencies of IL-17^+^ cells were found in IL-23-sufficient *Rag1^−/−^* hosts (data not shown). Moreover, the total amounts of IL-17 were not significantly increased in the colons of mice transferred with wild-type or Foxp3-deficient naive T cells (Figure 5B). Together, these data argue against an abnormal development of Th17 cells in the absence of functional Foxp3 in this system and point to a direct role of Foxp3 in inhibiting colitis in the absence of IL-23.

### Inflamed Colons Exhibit a Similar Cytokine Pattern in the Presence or Absence of IL-23

**Figure 6:**
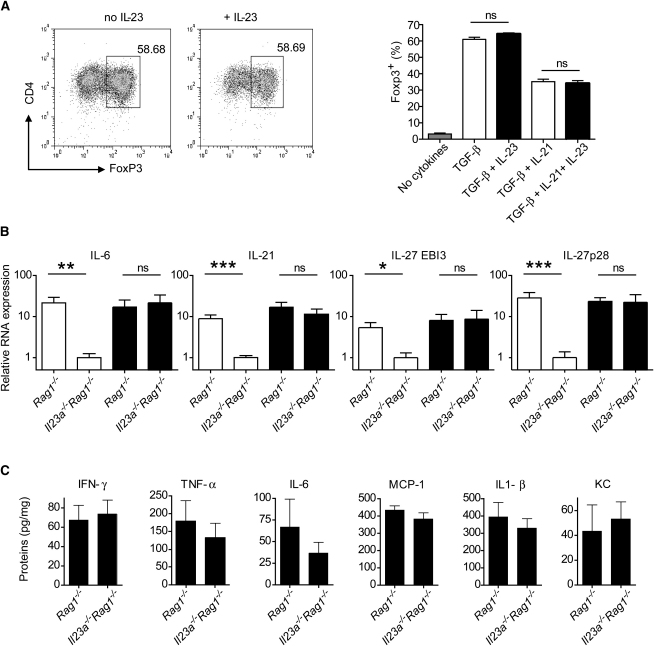
Cytokine Expression in Inflamed Colons from *Il23a^−/−^Rag1^−/−^* Mice (A) Foxp3 expression after culture of CD4^+^CD45RB^hi^ T cells with TGF-β, IL-23, and/or IL-21. Left: Representative FACS plots. Cells are gated on forward and side scatter to exclude dead cells. Right: Percentage of Foxp3^+^ cells in the CD4^+^ population. Data show mean + SEM of three replicates and are representative of three independent experiments. ns, not significant. (B) Relative mRNA expression of IL-6, IL-21, IL-27p28, and EBI3 in colon homogenates after naive T cell transfer with wild-type (empty columns) or dnTGFβRII or *Foxp3^−/−^* CD4^+^ T cells (filled columns). Data from mice transferred with dnTGFβRII or *Foxp3^−/−^* T cells were pooled because they yielded similar values. Data were normalized to HPRT for each sample. The average value for *Il23a^−/−^Rag1^−/−^* mice transferred with wild-type T cells (noninflamed) was set as one. Data show mean + SEM of between seven and 11 mice per group. ^∗^, p < 0.05; ^∗∗^, p < 0.01; ^∗∗∗^, p < 0.001; ns, not significant. (C) Amounts of proinflammatory cytokines in colon homogenates of *Rag1^−/−^* or *Il23a^−/−^Rag1^−/−^* transferred with *Foxp3^−/−^* naive T cells. Data show mean + SEM of between five and ten mice from two independent experiments. None of the differences were significant.

IL-6 and IL-21, two cytokines that promote Th17 cell polarization, have been described as directly blocking Foxp3 induction on T cells, and in vivo IL-6 blockade increases the frequency of Foxp3^+^ cells after naive T cell transfer (Figure S1 available online), suggesting that IL-23 and IL-6 could be acting in similar ways (Bettelli et al., 2006; Korn et al., 2007; Mangan et al., 2006; Nurieva et al., 2007; Zhou et al., 2007). However, unlike IL-6 and IL-21, IL-23 addition did not inhibit TGF-β-mediated Foxp3 induction on naive T cells in vitro (Figure 6 and data not shown), suggesting that the regulation of Foxp3 by IL-23 may be more complex than the direct inhibition previously described for other cytokines.

We next attempted to identify downstream effectors of IL-23-mediated inflammation. IL-23 could be indirectly regulating Foxp3 by controlling other cytokines. Indeed, colons from naive T cell-transferred IL-23-deficient mice exhibited markedly reduced amounts of several proinflammatory molecules, including IL-6 (Hue et al., 2006). However, it is difficult to distinguish whether this is a primary or a secondary effect because of the lack of inflammation. When we measured mRNA levels in inflamed colons from *Il23a^−/−^Rag1^−/−^* mice (Figure 6B, mice transferred with dnTGFβRII- or Foxp3-deficient T cells), IL-6 and IL-21 were found to be upregulated to similar values as in IL-23-sufficient *Rag1^−/−^* hosts, suggesting that the effect of IL-23 is independent of these cytokines. A similar pattern was found for the two subunits of IL-27 (p28 and EBI3), another cytokine inhibiting Foxp3 induction in vitro (Korn et al., 2007; Neufert et al., 2007). Together, the results indicate that IL-23 is not essential for the upregulation of IL-6, IL-21, and IL-27, which can themselves inhibit Foxp3 induction.

Importantly, IL-23-deficient recipients transferred with Foxp3-deficient naive T cells expressed similarly elevated amounts of TNF-α, IFN-γ, IL-6, IL-1β, KC, and MCP-1 proteins in the colon as control *Rag1^−/−^* recipients (Figure 6C). The increase in TNF-α and IFN-γ is especially relevant because these mediators have previously been shown to be essential for the disease induction in the naive T cell transfer model (Singh et al., 2001; Yamamoto et al., 2000). Hence, if immunosuppressive pathways are restrained, IL-23 is not required for upregulation of the inflammatory cytokines that induce colitis.

## Discussion

The immune response in the intestine is a delicate balance between effector and regulatory T cell responses. Recent studies have shown that IL-23 plays a key role in this balance and is a necessary factor for the development of T cell-dependent and -independent chronic intestinal inflammation (Elson et al., 2007; Hue et al., 2006; Kullberg et al., 2006; Uhlig et al., 2006b; Yen et al., 2006). The resistance of IL-23-deficient mice to colitis has been attributed to a reduction in pathogenic T cell responses, particularly those mediated by Th17 cells. Yet, in accordance with a previous report, we find that IL-17 production is not required by T cells to induce colitis (Noguchi et al., 2007). In addition, the absence of IL-23 did not significantly alter the intestinal amounts of IL-17 or the relative expression of RORγt in this model, suggesting that IL-23 can promote intestinal inflammation independently of its role in promoting Th17 cells. We show here that colitogenic T cell responses are retained in the absence of IL-23 but are masked by dominant IL-10- and TGF-β-mediated suppression. Furthermore, transfer of naive T cells to *Il23a^−/−^Rag1^−/−^* mice fails to elicit colitis and is associated with an increase in the frequency of CD4^+^Foxp3^+^ cells in the intestine. These Foxp3^+^ cells appear to play a functional role in protection from IL-23-independent inflammation because transfer of Foxp3-deficient T cells to *Il23a^−/−^Rag1^−/−^* hosts induces severe colitis, indistinguishable from disease induced after T cell transfer into IL-23-sufficient *Rag1^−/−^* recipients. These results newly identify an important role for IL-23 in restraining local Treg cell responses in order to permit development of tissue inflammation.

An important concept to emerge from these studies is that factors may promote inflammation not only via direct effects on inflammatory mediators but also indirectly by impeding regulatory mechanisms. Precedence for this idea comes from studies on the role of IL-6 in pathogenic Th17 cell responses in the central nervous system. Originally thought to be required for the development of Th17 cell responses, it was recently shown that IL-6 promotes Th17 cell responses in part by alleviating Treg cell-mediated control (Korn et al., 2007). Our data strongly suggest that IL-23 could be playing a similar role in the intestine. The fact that Foxp3-deficient T cells can induce high levels of colitis in the absence of IL-23 provides direct evidence that IL-23 is not essential to the pathogenesis of intestinal inflammation, if regulation is absent. Similarly, a number of key proinflammatory cytokines, including IL-6, IFN-γ, and TNF-α, can be expressed in the intestine in the absence of IL-23. Currently, it is not known whether the effects of IL-23 are mediated directly on T cells or act via effects on non-T cells that produce factors that inhibit Treg induction. IL-23 has been suggested to act directly on T cells to inhibit Foxp3 expression (Zhou et al., 2007). However, our in vitro experiments did not show a direct effect of IL-23 on TGF-β-mediated Foxp3 induction. It must be borne in mind that the effects of IL-23 seem specific to the intestine and could therefore require a particular environment different from conventional cell-culture conditions. Alternatively, IL-23 could have an indirect effect on Treg cell generation. Several cytokines have been described to directly inhibit Foxp3 induction, including IL-6, IL-21, and IL-27 (Bettelli et al., 2006; Korn et al., 2007; Mangan et al., 2006; Nurieva et al., 2007; Zhou et al., 2007). Although these cytokines could be involved in the control of Foxp3 generation by IL-23, IL-23 is not essential for their expression, because they are upregulated in inflamed colons from IL-23-deficient recipients. This suggests that IL-23 represents a distinct tissue-specific pathway to control Treg cell induction. Experiments with cell type-specific deletions of the IL-23R are required to dissect the individual components of this pathway in vivo.

By controlling Foxp3-mediated regulation, IL-23 may affect other pathways than Th17 cell responses. Because of the role of IL-23 in promoting IL-17 production, Th17 cells have been considered to play an important role in IL-23-dependent pathologies. This, however, may not be true in all cases. Although our data do not exclude the possibility that IL-23 directly sustains Th17 cells, they offer an additional explanation for its proinflammatory effects. Defects in IL-23 or IL-23R could lead to an increase or decrease in immune suppression that could affect not only Th17 cells, but also Th1 cells, Th2 cells, and innate immune responses. Indeed, a role for IL-23 in promoting non-Th17 cell responses has already been suggested by others. Thus, MOG-specific T cells from *Il23a^−/−^* mice have reduced amounts of IFN-γ, and systemic IL-23 can enhance Th1 cell antitumor responses (Kaiga et al., 2007; Thakker et al., 2007). Interestingly, both IL-23-deficient and -sufficient colitic mice showed increased amounts of intestinal IFN-γ, indicating a strong local Th1 response. CD4^+^ T lymphocytes and non-T cells have been identified as sources of intestinal IFN-γ in different models of colitis (Hue et al., 2006; Uhlig et al., 2006a). Indeed, the Th1 response has been shown to be involved in the pathogenesis of T cell transfer-mediated colitis and both IFN-γ and the Th1 cell-specific transcription factor T-bet play functional roles (Neurath et al., 2002; Powrie et al., 1994). Similarly, CD in humans has been linked to exacerbated Th1 cell responses (Cobrin and Abreu, 2005). IL-12 is known to play a pivotal role in the control of Th1 cell responses. However, its requirement for the development of inflammation varies depending on the model. By contrast with chronic models, acute inflammation linked to intestinal injury is IL-12-dependent and inhibited by IL-23 (Becker et al., 2006). Clearly, further studies are required to fully characterize the contributions of these two cytokines to intestinal inflammation in different models and in particular in human disease. Nevertheless, by controlling Foxp3, IL-23 could be permissive for the development of both Th1 and Th17 cell responses.

Strikingly, IL-23 reduced the percentage of naive T cell-derived Foxp3^+^ but had little impact on the frequency of the progeny from already developed Treg cells. Foxp3^+^ Treg cells can develop in the thymus (the so-called naturally arising Treg cells), but also be induced in the periphery. Recent data from our group and others have shown that Foxp3^+^ Treg cells can be induced in the intestine by a mechanism depending on TGF-β and retinoic acid (Coombes et al., 2007; Mucida et al., 2007; Sun et al., 2007). These Treg cells are induced by a specific subset of dendritic cells (DCs) expressing CD103, which is enriched in the GALT. In contrast to the CD103^+^ subset, CD103^−^ DCs isolated from the MLN do not induce Foxp3^+^ cells. Importantly, they express high amounts of IL-23p19 mRNA compared to the Treg cell-inducing CD103^+^ subset upon CD40 stimulation (Coombes et al., 2007). It is tempting to speculate that IL-23, together with TGF-β and RA, is one of several factors that decide whether a naive T cell will become regulatory and induce dominant tolerance toward its cognate antigen. Other factors could include Th1 cell-related cytokines. A recent report signaled no increase of CD4^+^CD25^+^Foxp3^+^ cells in *Il12b^−/−^ Ifng^−/−^* mice, which lack both IL-12 and IL-23 (Wang et al., 2007). The analysis was, however, performed on the spleen, not on intestinal cells, where we find the highest effect of IL-23. More research is required to elucidate the contribution of different cytokines to the homeostasis of Treg cells under steady-state conditions.

It should be noted that although immune regulatory pathways in the intestine prevent hyperreactivity toward dietary antigens and harmless commensal flora, they can prove a double-edged sword. It is imperative to the host to mount protective responses toward intestinal pathogens, and hence it is necessary to temporarily overcome dominant suppression and Treg cell activity. IL-6 has been identified as one such inflammatory mediator that desensitizes T cells to Treg cell-mediated suppression (Pasare and Medzhitov, 2003). IL-23, via its ability to impede Treg cell responses in the intestine, may promote host-protective immunity at this site. In support of this, IL-23-deficient mice, unlike wild-type mice, do not develop severe colitis after infection with the intestinal pathogen *C. rodentium*, but they fail to clear the bacteria and die within 2 weeks (Mangan et al., 2006).

In the years since the identification of IL-23, evidence has accumulated indicating that IL-23 orchestrates different aspects of the immune response in tissues. In addition to its proinflammatory action on the innate immune system and its proposed role in sustaining Th17 cell responses, we have identified the overcoming of Foxp3-mediated regulation as another key function of IL-23 during immune responses. Although the relevance of this mechanism in other experimental models and human disease remains to be ascertained, this could have important implications for both the understanding of mucosal immunity and designing therapeutic approaches to IL-23-dependent diseases.

## Experimental Procedures

### Mice

Wild-type C57BL/6, BALB/c, CD4-restricted dnTGFβRII C57BL/6, congenic B6.SJL-*Cd45*, C57BL/6 *Rag1^−/−^*, BALB/c *Rag2^−/−^*, C57BL/6 *Il17a^−/−^*, C57BL/6 *Il23a^−/−^ Rag1^−/−^*, and C57BL/6 *Foxp3^−/−^* mice were bred and maintained under specific pathogen-free conditions in accredited animal facilities at the University of Oxford. Experiments were conducted in accordance with the UK Scientific Procedures Act of 1986. Mice were negative for *Helicobacter* spp. and other known intestinal pathogens and were more than 6 weeks old when first used.

### Generation of Mixed Bone-Marrow Chimeras

Bone marrow isolated from 2- to 3-week-old B6.*Foxp3^−/−^* mice was depleted of T cells via anti-CD4 and anti-CD8 Abs together with anti-rat coated Dynabeads (Dynal). B6.Foxp3^−/−^ bone marrow was then mixed in a 1:1 ratio with bone marrow taken from B6.SJL-*Cd45* mice and injected intravenously into gamma-irradiated (5.5 Gy, 550 rad) B6 SJL CD45.1 mice. Eight weeks later, *Foxp3^−/−^* and wild-type naive T cells were sorted on the basis of expression of CD4, CD25, CD45RB, and CD45.2.

### Transfer of Naive CD4^+^CD45RB^hi^ T Cells

Naive CD4^+^CD45RB^hi^ T cells were isolated from spleens of C57BL/6 or C57BL/6 *Il17a^−/−^* mice via FACS sorting as previously described (Read et al., 2000). In brief, after enriching for CD4^+^ lymphocytes, single-cell suspensions were stained with PerCP-conjugated anti-CD4, PE-conjugated anti-CD25, and FITC–anti-CD45RB (all obtained from BD Biosciences). Naive CD4^+^CD25^−^CD45RB^hi^ T cells were purified (>99%) with a cell sorter (MoFlo; DakoCytomation). For isolation of cells from bone-marrow chimeras, cells were additionally stained with biotinylated anti-CD45.2 followed by streptavidin-APC (both from BD Biosciences). For some experiments, the CD4^+^ CD25^+^ CD45RB^low^ population was sorted and added in a 1:100 ratio to the naive population. Sex-matched RAG1^−/−^ recipient mice received 4 × 10^5^ CD4^+^CD45RB^hi^ T cells by intraperitoneal (i.p.) injection, and development of intestinal inflammation was monitored as described below.

### In Vivo Antibody Treatment

Anti-mouse IL-10R mAb (clone 1B1.2) (O'Farrell et al., 1998) and anti-mouse TGF-β1/2 (clone 1D11.16.8) (Dasch et al., 1989) were purified from hybridoma supernatant by affinity chromatography and shown to contain less than 1.0 endotoxin units per milligram of protein. Mice were injected i.p. with 0.5 mg of anti-IL-10R twice a week, or with 1 mg of anti-TGF-β three times a week, starting the day after the T cell transfer and lasting until the end of the experiment.

### Assessment of Intestinal Inflammation

Mice were killed when symptoms of clinical disease (significant weight loss or diarrhea) became apparent in control groups, usually around 8 weeks after initiation of experiments. Samples of proximal colon, mid-colon, and distal colon were immediately fixed in buffered 10% formalin. Four to five microns of paraffin-embedded sections were stained with hematoxylin and eosin, and inflammation was assessed with a modified version of a previously described scoring system (Read et al., 2000). Each sample was graded semiquantitatively from 0 to 3 for the four following criteria: degree of epithelial hyperplasia and goblet cell depletion; leukocyte infiltration in the lamina propria; area of tissue affected; and the presence of markers of severe inflammation such as crypt abscesses, submucosal inflammation, and ulcers. Scores for each criterion were added to give an overall inflammation score for each sample of 0–12. The total colonic score was calculated as the average of the individual scores from the sections of proximal colon, mid-colon, and distal colon. In the graphs shown, each point corresponds to an individual mouse. Micrographs show sections of mid-colon.

### Isolation of Leukocyte Subpopulations and FACS

Cell suspensions were prepared from spleen, MLN, and the LP as previously described (Uhlig et al., 2006a). The following antibodies were used for flow cytometry: anti-CD4 conjugated to PerCP or FITC, anti-mouse TCR-β conjugated to PE, biotinylated anti-CD45.2 (all from BD Biosciences), and anti-CD3 conjugated to Alexa 647 (eBiosciences). Biotinylated antibodies were detected with PerCP-conjugated streptavidin (BD Biosciences). For Foxp3 staining, cells were fixed in eBioscience Fix/Perm buffer after the surface staining, followed by permeabilization in eBioscience buffer and staining for Foxp3 conjugated to APC or FITC (eBioscience) according to the manufacturer's instructions. Cells were acquired with a FACSCalibur or FACSort (BD Biosciences) and analyzed with FlowJo (Tree Star). For staining of intracellular cytokines, cells were cultured for 4 hr as described (Uhlig et al., 2006a). After this in vitro stimulation, cells were stained for CD4 and then fixed in Fix/Perm buffer (eBioscience). This was followed by permeabilization in eBioscience buffer and staining with anti-IFN-γ APC, anti-IL-17 PE (all from BD Biosciences), anti-Foxp3-APC (eBioscience), or appropriate isotype controls (BD Biosciences).

### In Vitro Foxp3 Induction

Sorted CD4^+^ CD25^−^ CD45RB^hi^ T cells from C57BL/6 mice were resuspended in complete RPMI 5% FCS and incubated at 2.5 × 10^5^ cells/ml in the presence of Dynabeads Mouse CD3/CD28 T cell expander (Dynal, 2 μl/ml) and in the presence of TGF-β (R&D, 1 ng/ml), IL-23 (eBioscience, 10 ng/ml), and/or IL-21 (R&D, 50 ng/ml). Foxp3 expression was assessed by FACS after 72 hr.

### Quantitation of Cytokine Amounts in Intestinal Tissues

Frozen colonic tissue samples were processed as described (Hue et al., 2006). Sequences for primers sets and probes are described in the Supplemental Data. Protein concentrations were measured either with the cytometric bead assay (BD Biosciences) (IFN-γ, IL-6, TNF-α, MCP-1) or with the Luminex 100 assay (Bio-Rad Laboratories) (IL-1β, IL-17, KC), as described (Hue et al., 2006).

### Statistics

The nonparametric Mann-Whitney test was used for comparing pathology scores and data from colon homogenates, and an unpaired t test was used to examine percentages of Foxp3^+^, IFN-γ^+^, and IL-17^+^ cells. Differences were considered statistically significant when p < 0.05.

## Supplemental Data

Document S1. Primers, and Probe Sequences, and One Figure
