# Supplementary material for: Interleukin-23 Restrains Regulatory T Cell Activity to Drive T Cell-Dependent Colitis
Source: Immunity. 2008 Apr 11;28(4):559–70. doi: 10.1016/j.immuni.2008.02.019 (PMC2292821; doi:10.1016/j.immuni.2008.02.019)
Supplement: Document S1. Primers, and Probe Sequences, and One Figure [file mmc1.pdf]

Immunity, Volume *28*

## Supplemental Data

# Interleukin-23 Restrains Regulatory T Cell Activity

# to Drive T Cell-Dependent Colitis

**Ana Izcue, Sophie Hue, Sofia Buonocore, Carolina V. Arancibia-Cárcamo, Philip P. Ahern, Yoichiro Iwakura, Kevin J. Maloy, and Fiona Powrie**

Supplemental Experimental Procedures:

Primers and Probe Sequences for Taqman qPCR

| | Primers | Probe |
|---|---|---|
| HPRT | 5’-GACCGGTCCCGTCATGC-3’<br>and 5’-TCATAACCTGGTTCATCATCGC-3’ | 5’-ACCCGCAGTCCCAGCGTCGTC-3’ |
| CD3γ | 5’-TTACAGAATGTGTGAAAACTGCATTG-3’<br>and 5’-CACCAAGAGCAAGGAAGAAGATG-3’ | 5’-ACATAGGCACCATATCCGGCTTTATCTTCG-3’ |
| RORγt | 5’-CCGCTGAGAGGGCTTCAC-3’<br>and 5’-TGCAGGAGTAGGCCACATTACA-3’ | 5’-AAGGGCTTCTTCCGCCGCAGCCAGCAG-3’ |
| IL-6 | 5’-GAGGATACCACTCCCAACAGACC-3’<br>and 5’-AAGTGCATCATCGTTGTTCATACA-3’ | 5’-CAGAATTGCCATTGCACAACTCTTTTCTCA-3’ |
| IL-21 | 5'-ATCCTGAACTTCTATCAGCTCCAC-3'<br>and 5'-GCATTTAGCTATGTGCTTCTGTTTC-3' | 5’-AAGCCATCAAACCCTGGAAACAATAAGACA-3’ |
| IL-27p28 | 5’-ATCTCGATTGCCAGGAGTGA-3’<br>and 5’-GTGGTAGCGAGGAAGCAGAGT-3’ | 5’-TTCCCAATGTTTCCCTGACTTTCCA-3’ |
| IL-27EBI3 | 5’-GCCATGCTTCTCGGTATCC-3’<br>and 5’-GAGCCTGTAAGTGGCAATGA-3’ | 5’-TCCAGGCTCCCAACTCCACCA-3’ |

All probes were labeled FAM/TAMRA.

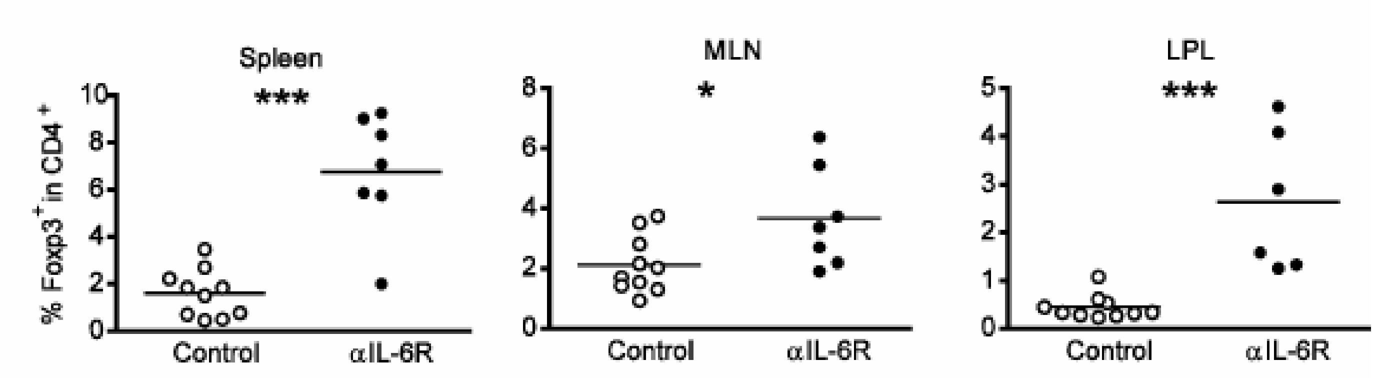


**Figure S1. Frequency of Foxp3$^+$ Cells among CD4$^+$ Cells after anti-IL-6R Treatment**

Frequency of Foxp3$^+$ among CD4$^+$ cells from spleen, MLN and colonic LPL from *Rag*$^{-/-}$ mice transferred with CD4$^+$ CD25$^-$ CD45RB$^{hi}$ naive T cells. Controls were left untreated, and the anti-IL-6R group received 2 mg of 15A7 purified antibody i.p. on the day of T cell transfer and then once a week until the end of the experiment. This treatment abrogated colitis as described (Yamamoto et al., 2000). Each point represents an individual mouse; data are pooled from two independent experiments, one on BALB/c *Rag2*$^{-/-}$ and another one on C57BL/6 *Rag1*$^{-/-}$ background which yielded similar results.
